# Messaging and Chatbot-Assisted Nursing Consultation (ChatACP) to Empower Family Members of Residents of Nursing Homes on Advance Care Planning: Protocol for a Mixed Methods Pilot Study

**DOI:** 10.2196/94883

**Published:** 2026-08-12

**Authors:** Tongyao Wang, Ho Nee Chu, Pui Hing Chau, Xue Zhou, Chia-Chin Lin

**Affiliations:** 1 School of Nursing, Faculty of Medicine University of Hong Kong Hong Kong China (Hong Kong); 2 Helping Hand Hong Kong China (Hong Kong)

**Keywords:** advance care planning, chatbot, family empowerment, generative artificial intelligence, generative AI, end of life, large language model, artificial intelligence, AI

## Abstract

**Background:**

Advance care planning (ACP) involves proactive communication about end-of-life care preferences among patients, families, and health care providers. In Chinese culture, older adults commonly delegate such care decisions to adult children, yet family reluctance—rooted in beliefs that aggressive treatments are beneficial—remains a major barrier to ACP participation. Existing interventions improve documentation and communication through education, hypothetical scenarios, and physician engagement, but few target family members specifically or use a theoretical framework. The transtheoretical model is particularly underused for assessing family readiness. Infographics, videos, and large language models demonstrate strong potential for engagement, highlighting the need for tailored, theory-driven family empowerment interventions.

**Objective:**

This study aims to evaluate the feasibility, acceptability, and preliminary efficacy of a 10-day digital intervention (ChatACP) designed to empower family members to support care home residents in ACP.

**Methods:**

An explanatory sequential mixed methods study using a pilot randomized controlled trial with a postintervention and 3-month assessment and a posttrial qualitative study will be conducted. Eligible family members (N=60) of care home residents from 6 homes will be enrolled. The intervention group will receive ChatACP, and the control group will receive a self-learning ACP pamphlet. The ChatACP intervention consists of 2 phases. Phase 1 includes a 10-day series of ACP educational infographics, video clips, and a content-specific chatbot on ACP knowledge in Hong Kong. Upon completion of phase 1, those not advancing to the action stage will receive phase 2 of a nurse-led telephone consultation.

**Results:**

This project was recommended for support in September 2025. Recruitment and data collection will commence in September 2026. Primary outcomes of feasibility and acceptability include rates of recruitment, retention, fidelity, engagement, usability, and safety. Secondary outcomes include the family members’ ACP readiness, engagement, and completion of ACP activities. The feasibility and acceptability outcomes will be compared to predefined progress criteria. Preliminary efficacy outcomes will be analyzed using a generalized mixed-effects model.

**Conclusions:**

The study will inform whether the ChatACP intervention can empower families on ACP. Findings from this study can inform the development of large-scale interventions to improve ACP engagement among family members of care home residents.

**Trial Registration:**

ClinicalTrials.gov NCT07448649; https://clinicaltrials.gov/study/NCT07448649

**International Registered Report Identifier (IRRID):**

PRR1-10.2196/94883

## Introduction

Advance care planning (ACP) is “an overarching process of proactive communication regarding end-of-life care” among patients, family caregivers, and health care providers [1]. Unlike Western conventions, Chinese parents often rely on their children for emotional and financial security and care decision-making in their later years, assuming that it is a natural outcome of the sacrifices and resources they invested in raising their children [2]. However, one randomized controlled trial (RCT) found family objections a key barrier to older individuals’ participation in ACP activities [3]. Given family members’ reluctance to discuss their loved ones’ end-of-life (EOL) care preferences, stemming from the belief that aggressive therapies are the most beneficial, interventions are necessary to empower them to alter their perspective.

A meta-synthesis of 17 qualitative studies [4] on family members’ experiences with ACP in nursing homes revealed that family members lacked knowledge and awareness of the timing and goals of ACP. In addition, there were 28 studies, including 14 RCTs and 14 nonrandomized experimental studies retrieved from both Chinese- and English-language databases [5,6], that demonstrated effectiveness in completing advance medical directive documentation (odds ratio [OR] 7.58, 95% CI 1.41-40.63; *P*=.02) and proactive communication with physicians (OR 2.42, 95% CI 1.42-4.12; *P*=.001). A total of 20 studies [5,6] adopted ACP education interventions (information manual, videos, goal-of-care statement, and interactive websites) in promoting ACP awareness. Trained nurses, social workers, physicians, or trained laypeople delivered 11 to 120 minutes of ACP consultations. Additional components focused on engagement with care providers. Four previous studies examined the use of hypothetical stories to have participants reflect from a third-person point of view [7-10]. Group visits, mindfulness activities, and card games have introduced EOL topics in a supportive atmosphere [11-13]. One study investigated a coaching session on asking care providers about EOL care preferences [14]. Thus, for an ACP empowerment intervention, ACP education and care provider engagement are 2 key components.

None of the existing interventions have reported detailed information on how to empower family members or are guided by a conceptual framework. While the transtheoretical model (TTM) has been widely used to understand the stages of change (precontemplation, contemplation, preparation, action, and maintenance stages) for ACP among older adults [15], its potential for informing and enhancing social and behavioral interventions for family members remains underused [16]. The Hong Kong Hospital Authority recommended that ACP should be sensitive to assessing the readiness of the patient and family members to continue the conversations and a rigid or routinized approach should be avoided [1]. Therefore, there is a pressing need for research that investigates the application of the TTM in designing targeted ACP empowerment interventions for family members.

Compelling evidence supports infographics and videos for patient education as they also lower patient anxiety [17,18]. Large language models (LLMs) such as the generative pretrained transformer series and DeepSeek have demonstrated capabilities in answering queries when trained using targeted content in a user-friendly language, and they show significant promise for enhancing patient engagement [19,20]. To empower family members to participate in their loved ones’ ACP, our team has developed the evidence-based, theory-driven ChatACP intervention. ChatACP leverages the strengths of infographics and video instruction, tailoring them to family members in the precontemplation and contemplation stages while also providing interaction with an LLM-based chatbot specializing in ACP and consultation with a research nurse for those in the preparation stage.

## Methods

### Aims

The study aims to evaluate (1) the feasibility and acceptability of ChatACP and (2) the preliminary efficacy of ChatACP on family members’ readiness for ACP, engagement in ACP, and completion of ACP activities, in comparison to those who received a self-learning handout, at the postintervention time point and 3-month follow-up.

### Design

**Figure 1:**
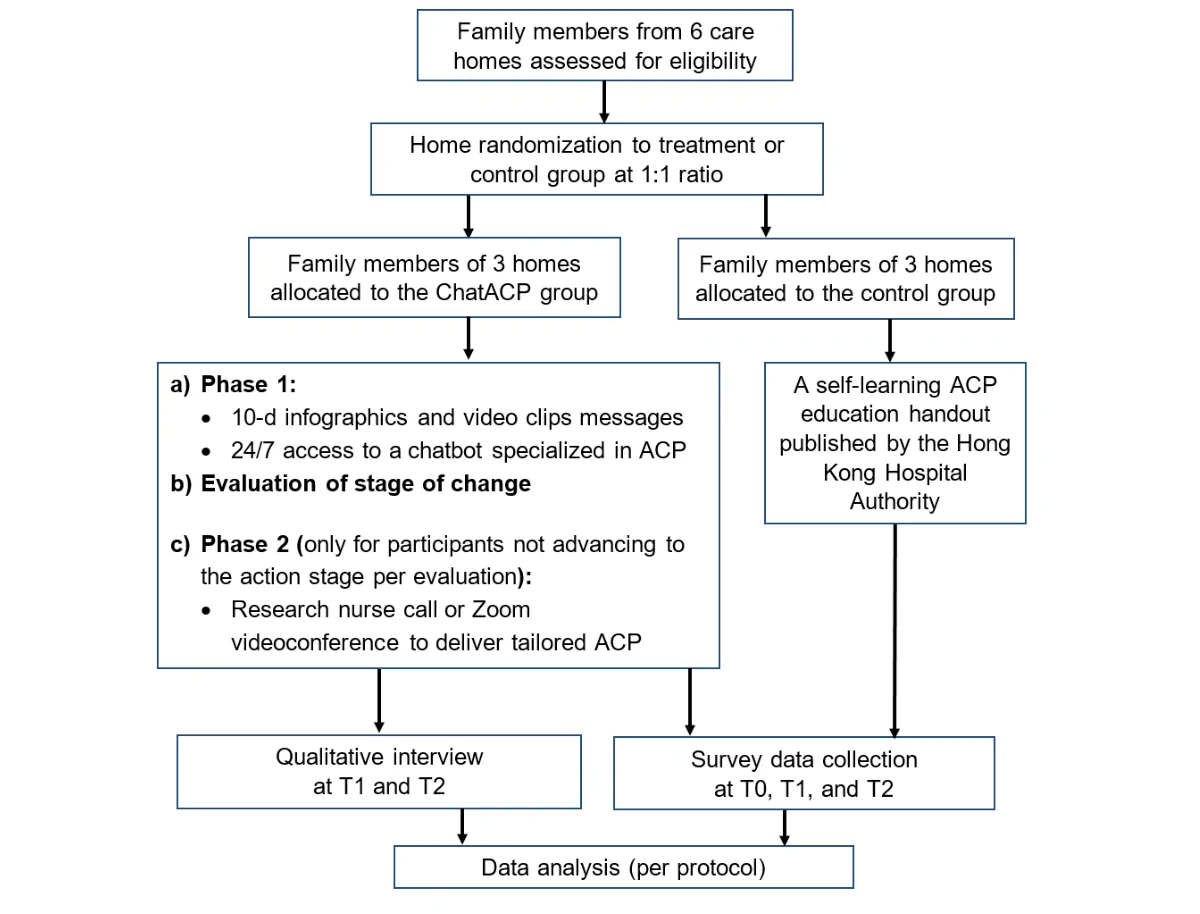
Trial flowchart. ACP: advance care planning; T0: baseline; T1: postintervention time point; T2: 3-month follow-up.

This will be an explanatory sequential mixed methods study including a pilot cluster RCT followed by a posttrial qualitative study with a postintervention time point and 3-month follow-up, as shown in the trial flowchart (Figure 1), over 2 years. To prevent contamination, we will enroll 6 care homes (N=60 family members of residents) and randomly assign 3 homes to receive ChatACP and 3 to receive the ACP self-learning handout.

### Inclusion Criteria

**Figure 2:**
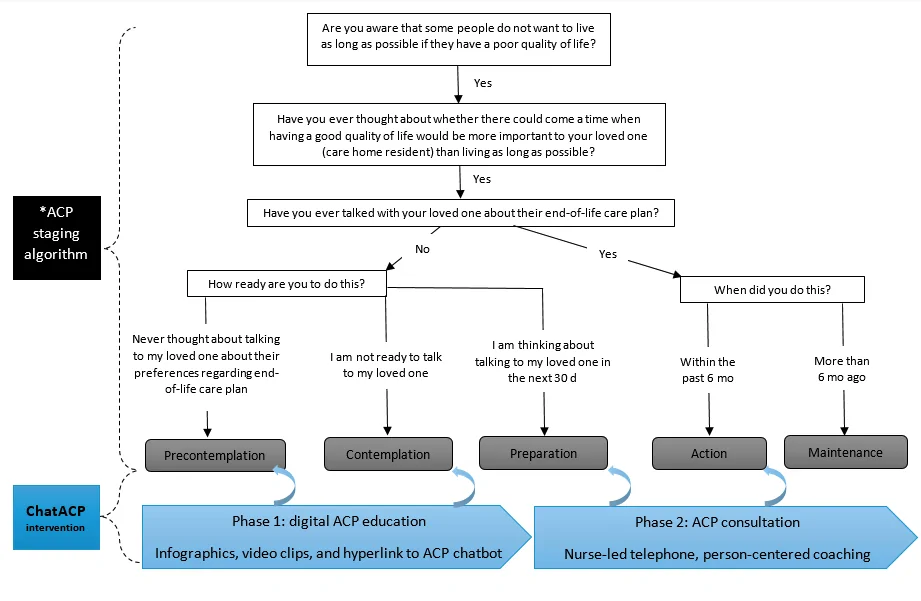
Theoretical framework for the ChatACP intervention based on the advance care planning (ACP) staging algorithm (adapted from Fried et al [22]).

The inclusion criteria are as follows: (1) age of 18 years or above; (2) providing care for an older adult (aged 65 years or older) living in a care home as a primary family caregiver (including family members in the traditional sense and the guardians and persons close to or significant to the older adult); (3) ability to read, write, and communicate in Cantonese or Mandarin; (4) the resident (care recipient) having a prefrail or frail status as defined by a score of 2 or above on the FRAIL scale [21]; (5) having a mobile device to receive instant messages and access to a cellular network; and (6) having engaged in minimal or no prior EOL care discussions with the care home resident as defined by a self-reported status of being in the precontemplation or contemplation stage regarding ACP discussions with the resident using the ACP staging algorithm illustrated in Figure 2 [22].

### Exclusion Criteria

The exclusion criteria are as follows: (1) moderate to severe cognitive impairment, defined by an Abbreviated Mental Test score [23] of 5 or less, and inability to give consent; and (2) communication problems (eg, deafness or aphasia).

### Randomization

The care home will be the unit of randomization, and 6 homes will be pair matched based on type of home (public or private). An independent statistician will randomly assign one home from each pair to the intervention arm using a computer-generated algorithm.

### Treatment

#### Overview

Prior to treatment allocation, all participants will be in the precontemplation or contemplation stage. Family caregivers in the clusters of the intervention group will receive the ChatACP intervention. Family caregivers in the clusters of the control group will receive a self-learning ACP handout published by the Hong Kong Hospital Authority [24].

#### ChatACP Group

##### Overview

ChatACP encompasses a digital education component and a care provider engagement component. The digital education component (messages with ACP infographics and video clips and a chatbot with specific content for ACP for 10 days) was developed to help family members in the precontemplation or contemplation stage in progressing to the preparation stage by enhancing their understanding of the value of ACP discussions. The care provider engagement component (nurse-led telephone consultation) was designed to coach family members in the preparation stage to advance to the action stage by formulating an action plan for initiating ACP with their loved ones.

##### Phase 1 (Digital Education)

Participants will receive the ChatACP education component (ACP infographics and video clips and access to a 24/7 ACP chatbot for 10 days) delivered via their preferred messaging platform (eg, WhatsApp, WeChat, or Line) by the research nurse. Participants will also be advised to submit their ACP queries through the chatbot or by messaging the research nurse. The nurse will respond to queries during working hours. Participants will be encouraged to share the content with the resident and other family members. To safeguard participant privacy, each participant will be assigned a unique study ID for identification, and participants will be instructed to avoid disclosing any personally identifiable information on the messaging platform or chatbot. All the chatbot and message logs will be collected and securely saved in a cloud drive at the University of Hong Kong. Access to the data is restricted solely to the research team, and no external platforms are permitted access.

ACP infographics (Table 1) were developed for care home settings by incorporating ACP teaching materials from Singapore, the United Kingdom, the United States, and Canada. The main content consists of educational materials on EOL care, hypothetical scenarios, and communication skills. A panel of local health professional experts and laypersons appraised the ACP content and provided feedback as well. We tailored the ACP infographics according to the TTM of readiness to change, adjusting the content from simple to advanced. These infographics were segmented into a 10-day delivery format.

ACP video clips were also developed based on the 10-day infographic protocol. A total of 10 videos of 1 to 2 minutes with infographics, animated illustrations, subtitles, and audio were made by our team using artificial intelligence–powered visual communication tools.

The ACP chatbot was developed on the cutting-edge open-source LLM DeepSeek-R1 [25], which ensured the baseline conversational intelligence, and then customized to have ACP expertise by feeding it specific knowledge compiled by our team. The database includes local ACP guidelines and a document including frequently asked questions and answers gathered from older adults, family caregivers, and care providers from care homes. The ACP chatbot was also designed to draft responses at a fifth-grade reading level. The ACP chatbot functions as a search engine specialized in ACP; family members can access the chatbot via a web link 24/7. The accuracy of the chatbot will be evaluated internally prior to deployment for participant use. The chatbot has been prompted to not provide clinical advice and to direct participants expressing distress to contact the research nurse for immediate support during working hours (9 AM to 6 PM, Monday to Friday). The research nurse is an experienced Respecting Choices–certified ACP facilitator who has successfully managed over 100 cases of ACP for older adults and their families in Hong Kong. All the conversations within the chatbot will be reviewed by the research assistants (RAs) for accuracy and appropriateness daily during the intervention period. In the event of detecting inaccurate or improper responses from the chatbot, RAs will escalate the case to the research nurse to directly approach the participants via the messaging platform to provide clarification on the chat content.

**Table 1:** ChatACP educational infographic messaging protocol.

Day	Topic of the infographics
1	What is advance care planning? Disease trajectories at the end of life Hypothetical scenarios and guidance for common family member issues
2	Why plan ahead? Hypothetical scenarios and guidance for common family member issues
3	First step: understanding the patient’s needs and wishes
4	Second step: the timing of communication with family members
5	Third step: communicating with the physician
6	The role of the family member as a spokesperson for medical decision-making
7	Life-prolonging treatments, including cardiopulmonary resuscitation, assisted ventilation, artificial nutrition and liquid feeding, blood transfusions, and dialysis
8	What is an AMD^a^? Points to note when signing an AMD Hypothetical scenarios and common issues faced by family members
9	How can family members initiate conversations about end-of-life care with the patient? Comfort-oriented treatment Hypothetical scenarios and guidance on common issues faced by family members
10	What can family members do for patients who are terminally ill?

^a^AMD: advance medical directive.

##### Interim ACP Staging Assessment

Participants will be asked to self-report their stage of change. Those in the action stage will be exempt from entering phase 2 of the intervention. Those not in the action stage will receive nurse-led ACP consultations in phase 2.

##### Phase 2 (Care Provider Engagement)

Following the digital education component, if participants continue to indicate unpreparedness for discussing EOL topics, the research nurse will offer tailored ACP consultation. Depending on their level of readiness, the research nurse will coach them on how to initiate ACP activities with their loved ones. The trained research nurse will reach out to participants via phone to address any inquiries regarding ACP and encourage them via motivational interviewing skills.

### Outcome Measurements

#### Preliminary Efficacy Outcomes

The following measures will be collected among participants in both arms at baseline (T0; before the intervention), after the intervention (T1), and at 3 months (T2).

#### Family Members’ ACP Readiness

The measurement consists of 10 items rated on a 7-point Likert scale on ACP knowledge, readiness to discuss ACP with a loved one, self-efficacy in initiating ACP conversations with a loved one, barriers to such initiation, and emotional perceptions of and attitudes toward ACP, as well as whether family members have the necessary communication tools for ACP conversations. This instrument has been validated in Chinese (Cronbach α=0.86-0.90) [26].

#### Caregiver-Reported ACP Engagement

The ACP engagement survey for surrogate decision-makers (SDMs) comprises 17 items rated on a 5-point Likert scale [27]. It measures the surrogate’s engagement in the ACP process, with 3 domains including serving as an SDM (7 items), contemplation (4 items), and readiness (6 items). It has been validated in surrogates of patients with chronic illnesses [27], and the Chinese version has high internal consistency (Cronbach α=0.90-0.91) [28].

#### Completion of ACP Activities

Participants will report a binary response of “yes” or “no” on six behaviors widely used to evaluate ACP outcomes [15]: (1) complete the designation of a health care proxy for the resident; (2) communicate with the resident about their views on life-sustaining treatment and (3) quality vs quantity of life; (4) help residents’ communicate with their health care providers regarding resident’s views on the use of life-sustaining treatment and (5) quality vs quantity of life; and (6) help the resident complete their EOL care paperwork, such as documenting ACP in health medical records and advance medical directives or do-not-attempt-cardiopulmonary-resuscitation forms.

#### Feasibility and Acceptability Outcomes

##### Recruitment Feasibility

Recruitment rate will be assessed by calculating the percentage of eligible family members who provide study consent and actively participate in the study.

##### Retention Rate

This will be determined through the proportion of participants who successfully complete all the follow-up surveys.

##### Intervention Fidelity

This will be evaluated through the compliance rate with the intervention protocol during intervention delivery by the RA and research nurse.

##### Intervention Engagement

Engagement will be measured by checking the conversation log automatically recorded by the messaging platform and the chatbot. Participants will also report the extent to which they have read the daily messages during the intervention period (0%=did not read them at all; 100%=read all the messages).

##### Usability of the Intervention Content

The appropriateness of the content will be assessed using the perceived infographic usability measurement [29], which evaluates message-delivered infographics on trustworthiness, clarity, risk of confusion, difficulty to follow, level of usefulness, informativeness, comprehensibility, and simplicity. Each item will be scored on a sliding scale from 0% to 100%, with 0% indicating that there is no presentation of the above characteristics and 100% indicating that there is a presentation of 100%.

##### Intervention Acceptability

This will be measured via an investigator-developed satisfaction survey in terms of form of delivery, availability of customized support on ACP questions, and degree to which the intervention met the participants’ needs on a 5-point Likert scale (1=“highly dissatisfied”; 2=“dissatisfied”; 3=“neither satisfied nor dissatisfied”; 4=“satisfied”; 5=“highly satisfied”).

##### Intervention Safety

Risk assessment will be conducted using self-reported adverse events.

##### Qualitative Interviews

An interview guide will be used to understand participants’ experience of the intervention, including the acceptability of the burden of the program and logistics, as well as how to further improve the intervention.

### Ethical Considerations

This study received ethics approval from the University of Hong Kong and Hospital Authority Hong Kong West Cluster ethics review board (UW25-567) in December 2025. Written informed consent will be obtained from all participants, ensuring their right to withdraw per the Declaration of Helsinki. Participant data will be deidentified, stored securely, and accessed only by authorized personnel to ensure privacy and confidentiality.

### Recruitment

Recruitment will be conducted in accordance with the protocol approved by the relevant ethics committee. Family members will be recruited from 6 care homes. All the eligible family members from the participating homes will be invited to join the study. Our RA will first contact the superintendent on their interest in joining the study. After obtaining agreement from the administration, we will promote the study in the homes during family events and using promotional posters. In addition, our RAs will work with the home staff (nurses, social workers, or other care providers) on identifying eligible family members. Staff at nursing homes will first obtain a verbal expression of interest from the family members before passing their contact information to our RA for recruitment. Regarding the qualitative interviews, family members of different sexes, relationships to the older adults, levels of intervention engagement, and motivation stages for ACP, as well as residential care home staff (approximately n=20 depending on the informational redundancy), will be invited for an individual semistructured interview upon completion of the intervention and at the 3-month follow-up.

### Data Collection

The preliminary efficacy outcomes will be collected among participants in both arms at baseline (T0; before the intervention), after the intervention (T1), and at 3 months (T2). The feasibility of the proposed study will be evaluated using the feasibility and acceptability outcomes at the end of data collection. Following the completion of the T2 survey, participants will receive HKD 200 (HKD 1=US $0.13 as of June 5, 2026) supermarket coupon as an incentive.

### Analysis

Data will be analyzed in SPSS software (IBM Corp). Data will be screened for completeness, and the RA will contact participants if there are any missing data. Descriptive statistics will be used to summarize participant characteristics and study outcomes.

The preliminary effect outcomes (family members’ ACP readiness, engagement, and completion of ACP activities) will be analyzed using a generalized linear mixed-effects model to understand the treatment differences at each time point. Because data are nested within each care home (cluster), observations within the same cluster may be correlated, violating the assumption of independence. To account for this clustering effect, we will integrate a random intercept for care homes in the generalized linear mixed-effects model. Given that our study is not powered to examine effectiveness, the outcomes will be reported with the standardized effect size (scores for ACP readiness and engagement) or ORs (completion of ACP activities) with a 95% CI. A Cohen *d* of 0.2, 0.5, and 0.8 and an OR of 1.5, 2.5, and 4 will indicate small, medium, and large effect sizes, respectively.

The feasibility and acceptability outcomes will be assessed to determine the progression to a full multisite RCT based on the predefined progression cutoffs outlined in the outcome measurements section, which include a 50% recruitment rate, 70% retention rate, 90% intervention fidelity, 50% intervention engagement rate, 78% content usability, 70% acceptability, and 100% safety based on prior review [30].

For the qualitative evaluation, the voice-recorded interviews will be transcribed verbatim and then analyzed according to the thematic analysis proposed by Braun and Clarke [31]. The NVivo software (Lumivero) will be used to manage the collected qualitative data and support the analysis. To ensure rigor, credibility, transferability, dependability, and confirmability will be assessed. Findings from both the qualitative and quantitative portions after completing the qualitative study will be synthesized. A sequential interpretive integration of both types of data will be undertaken at the interpretation stage.

## Results

This project was approved by the ethics review board on December 5, 2025 and was funded in May 2026. Recruitment and data collection will commence in September 2026. The results are expected to be published in September 2028.

## Discussion

### Expected Findings

This represents, to our knowledge, one of the first experimental studies to examine an LLM-enhanced ACP consultation, addressing a critical gap in tailoring the presentation of ACP content to a layperson-friendly literacy level. In addition, this study offers a potential solution to address the phenomenon of information overload [32] using LLM-based consultation. The potential application of findings from the ChatACP pilot study could have significant implications for patients, their families, and the health care system.

This protocol describes a pilot cluster RCT with an explanatory sequential mixed methods design to evaluate the feasibility, acceptability, and preliminary efficacy of ChatACP, a novel digital intervention designed to empower family caregivers of frail older adults in Hong Kong residential care homes to engage in ACP. ChatACP addresses key cultural barriers in Chinese contexts—where older adults often defer EOL decisions to adult children, yet families frequently resist open discussions due to preferences for aggressive treatment—by progressing caregivers along the TTM stages from precontemplation or contemplation toward preparation and action. The intervention combines a 10-day digital education package (culturally adapted infographics, short artificial intelligence–generated videos, and a 24/7 customized LLM-based chatbot) with optional nurse-led motivational interviewing consultations. The active control—a standard self-learning ACP handout from the Hong Kong Hospital Authority—represents usual care, enabling a pragmatic comparison of whether theory-driven, interactive digital support can yield superior outcomes in ACP readiness, engagement, and behavioral completion.

The cluster randomized design, with care homes as the unit of randomization and pair matching by public or private status, is a core strength that minimizes the contamination risks common in shared residential settings. With 6 clusters, the modest sample (N=60) is appropriate for a pilot focused on feasibility rather than definitive efficacy. The mixed methods approach further enhances rigor: predefined quantitative progression criteria (eg, recruitment of ≥50%, retention of ≥70%, fidelity of ≥90%, engagement of ≥50%, usability of ≥78%, acceptability of ≥70%, and safety of 100%) provide objective benchmarks for advancing to a full-scale multisite RCT, whereas postintervention qualitative interviews will capture nuanced experiences, perceived burden, logistical challenges, and suggestions for refinement. The intervention’s innovation lies in its integration of accessible, low-literacy digital tools tailored to TTM stages, including interim self-reported staging to trigger personalized phase 2 coaching. The use of validated Chinese-language measures (ACP readiness scale and ACP engagement survey for SDMs) and binary behavioral outcomes ensures culturally relevant, reliable assessment. The chatbot’s fine-tuning on local guidelines, stakeholder-informed frequently asked questions, and fifth-grade reading level responses offer scalable, on-demand support, aligning with calls for flexible, family-centered ACP approaches.

Despite these strengths, there are important limitations. As this is a pilot study, the small sample and limited number of clusters reduce statistical power and increase vulnerability to baseline imbalances or cluster effects. Generalizability remains restricted to Hong Kong care home contexts, where family dynamics, digital access, and institutional practices may differ from those in mainland China or other Asian settings. Self-reported outcomes, while validated, are prone to social desirability bias or recall inaccuracies, especially on sensitive EOL topics. Digital delivery requires smartphone ownership, network connectivity, and technological comfort, potentially excluding less technologically savvy caregivers and raising equity concerns in an aging population. Recruitment relies heavily on care home staff cooperation and family member availability, which can be unpredictable amid competing caregiving demands or resident health fluctuations.

These considerations highlight the pilot’s primary role: generating critical data on the operational feasibility, participant engagement with digital components, and cultural acceptability of an LLM-augmented intervention. If the progression criteria are achieved, the findings will guide protocol refinements—such as supplementary support for low-engagement users, expanded offline options, or additional content modules—for a larger trial powered to assess longer-term impacts on ACP documentation, provider communication, and concordance between care preferences and actual EOL care. By addressing family empowerment through innovative, theory-informed digital strategies, this work contributes to developing culturally congruent ACP interventions suited to resource-constrained care home environments in aging societies.

### Impact on Care Quality and Patient-Centeredness

The ChatACP intervention has the potential to improve the quality of care for care home residents by aligning medical and nursing care with residents’ preferences and values. By empowering family members to engage in ACP discussions, residents’ individual wishes can be better integrated into their health care plans, resulting in more patient-centered care. This approach may reduce unnecessary hospitalizations and invasive, often futile medical procedures while alleviating the emotional burden on family members who may otherwise struggle with decision-making during critical moments.

### Implications for Health Care Settings and Policy

By adopting this low-cost digital ACP empowerment intervention, nursing homes may reduce the burden on health care staff and streamline communication with family members. The findings may provide valuable insights into the use of chatbot technology in enhancing nursing consultations and promoting ACP engagement. If proven effective, the ChatACP intervention could be integrated into routine nursing practice in care homes and other health care settings, leading to increased ACP awareness and engagement among family members and health care professionals. This could foster a more proactive approach to ACP and EOL care. Additionally, the findings could inform policymakers about the potential benefits of incorporating messaging and chatbot systems into life-and-death education in nursing homes and guide the development of similar interventions for other aspects of health care management, such as chronic disease management or mental health support.

## Abbreviations

ACP: advance care planning
EOL: end of life
LLM: large language model
OR: odds ratio
RA: research assistant
RCT: randomized controlled trial
SDM: surrogate decision-maker
TTM: transtheoretical model
